# Patterns, circumstances and risk factors associated with non-fatal substance overdose in a cohort of homeless population: an observational study

**DOI:** 10.1007/s11096-024-01812-z

**Published:** 2024-11-19

**Authors:** Jennifer Anderson, Om Kurmi, Richard Lowrie, Adnan Araf, Vibhu Paudyal

**Affiliations:** 1 College of Medical and Dental Sciences, University of Birmingham, Birmingham, UK; 2 Centre for Healthcare and Communities, Coventry University, Coventry, UK; 3 Centre for Homelessness and Inclusion Health, School of Health in Social Science, University of Edinburgh, Edinburgh, UK; 4 Birmingham and Solihull Mental Health NHS Foundations Trust, Birmingham, UK; 5 Florence Nightingale Faculty of Nursing, Midwifery and Palliative Care, King’s College London, London, UK

**Keywords:** Drug overdose, Homelessness, Non-fatal overdose, Primary care, Substance overdose

## Abstract

**Background:**

Non-fatal overdoses frequently precede fatal overdoses, thus identifying risk factors for non-fatal overdoses could help develop strategies to prevent substance related deaths.

**Aim:**

This study aimed to identify patterns, circumstances and risk factors leading to non-fatal substance overdose in people experiencing homelessness.

**Method:**

All recorded cases of non-fatal substance overdose from a population of people experiencing homelessness registered at a specialist homelessness primary care centre in England were identified using electronic medical records. Overdose details and patient characteristics were extracted. The heterogeneity between variables in people with and without a recorded non-fatal overdose were tested and multivariable logistic regressions were used to identify the risk factors of non-fatal overdoses.

**Results:**

From the 1221 registered patients, 194(16%) were identified as having had a non-fatal overdose with 428 overdoses between them. Half were polypharmacy events with the main substances of overdose being: heroin, paracetamol, benzodiazepines, cocaine, antipsychotics, SSRIs and synthetic cannabinoids. Risk of non-fatal overdose was greater in females, white ethnicity, ages 36–45, and in those with a recorded use of tobacco, alcohol or illicit substance use. Chronic physical and mental health conditions increased the risk of non-fatal overdose including respiratory conditions, blood borne viruses, migraines, anxiety and depression.

**Conclusion:**

With a high number of non-fatal overdoses within this population, identifying individuals at risk based on the factors identified in this research could enable primary care providers to apply prevention actions such as overdose awareness and naloxone provision to avoid drug harm and deaths. Future work should explore the role of chronic physical conditions and their treatment on non-fatal overdose risks.

**Supplementary Information:**

The online version contains supplementary material available at 10.1007/s11096-024-01812-z.

## Impact statements

- Non-fatal overdose events recorded in primary care can enable service providers to offer prevention actions such as overdose awareness and naloxone provision to avoid future fatal events.
- Understanding of risk factors for non-fatal overdose in homeless populations identified in this study such as female sex, white ethnicity, history of alcohol and substance use disorders can allow targeted approaches for prevention actions.
- Addressing wider determinants of health such as physical, mental health, addiction, social and housing issues is key to preventing substance overdose.

## Introduction

Globally, substance overdose deaths remain a key public health concern. In England and Wales, a total of 4859 deaths related to substance poisoning were recorded in the year 2021, equivalent to 84.4 deaths per million people [1]. In particular, substance overdose was the most important cause of death among people experiencing homelessness (PEH), responsible for over a third of all deaths [2].

Rising homelessness and associated early deaths necessitate patterns and risk factors leading to overdose incidents in PEH to be better understood to strengthen prevention actions. In England alone, during 2020–21, 282,000 people were identified as homeless or threatened with homelessness, with numbers expected to increase further due to the current cost of living crisis [3]. PEH typically have a complex and unique set of medical and social needs, including increased early-onset age-related diseases [4], co-existing substance use and mental health disorders [5, 6] often, in part, a result of psychological and/or childhood trauma [7, 8].

Overdoses can be fatal or non-fatal (NFOD), with the latter a predicting factor of future fatal overdose [8]. PEH are at an increased risk of NFOD [9] and they are likely to have multiple overdoses, with one study reporting a mean of 3.2 NFODs over six months [10]. NFODs frequently require help from ambulances and/or present to emergency departments (ED) with significant cost implications to the health system and society [11–13]. Within England, substance overdose is known to account for 17.9% of all ED visits by PEH compared to only 1.9% of the general population with rates more than doubling in the last ten years [12].

NFODs are a strong predictor of subsequent overdose (fatal and non-fatal), and fatal overdose is the most common cause of death in PEH [14]. The importance of this public health epidemic of substance use disorder, overdose, and the resulting increased pressures on emergency services is emphasised by national initiatives that are encouraging innovative models of research and care [15, 16]. However, there is a dearth of descriptive or analytical studies of overdose in PEH, with the extent, nature and risk factors (e.g. sex, physical health conditions) for NFODs poorly understood.

### Aim

This study aimed to identify patterns, circumstances and risk factors leading to non-fatal substance overdose in people experiencing homelessness.

### Ethics approval

The study was reviewed and approved by institutional review board of Birmingham and Solihull Mental Health NHS Foundation Trust, approval reference number: 2023/SE0369.

## Method

Participants included all patients registered at a specialist homeless primary healthcare centre in the West Midlands region of England. The centre provides access to various healthcare professionals—GPs, nurses, and podiatrists—alongside psychotherapy and hosts a street outreach service. A substance dependence treatment centre is located in the city in a separate setting.

Data were collected using the electronic patient record system (EMIS Web). This system contains internal consultation and prescription records and external documents from other services (e.g. secondary care, and ambulance services). The reporting function was used to identify all non-fatal overdoses that had ever been recorded based on specified coded terms (Electronic Supplementary Material 1). The coded terms are set by the Systemised Nomenclature of Medicine Clinical Terms (SNOMED CT) and used throughout the National Health System (NHS) in the UK. Relevant codes are added to patient records by staff at the practice based on consultations and external documents received including ambulance and hospital discharge summaries. General characteristics were also extracted based on these coded items for all participants (age, gender, race, accommodation status) alongside terms to identify mental and physical health problems on record (Electronic Supplementary Material 2) and substance use (Electronic Supplementary Material 3).

Where a substance overdose was identified, medical records were searched for information regarding the overdose. This included documents from ambulance reports and hospitals (discharge letters from A&E and hospital wards, liaison psychiatry letters where relevant), free text searching to identify relevant consultation notes and free text searching within the ‘problems’ tab.

The following information was recorded for each overdose where available: number of overdoses for that individual, the substances overdosed on (including if alcohol was involved—Electronic Supplementary Material 4), ambulance attendance, administration of naloxone, ED attendance, hospital admissions and any suicidal intent. Free text information was also documented if relevant.

### Data analysis and statistics

The exported text data were changed to binary form using a custom MATLAB (R2023b) program. Statistical analysis was conducted using Stata version 16 to identify factors associated with NFOD. Differences in the characteristics of those with a NFOD and those without were determined using chi-square tests and Wilcoxon rank sum tests for normally and non-normally distributed data, respectively. Odds ratios and 95% confidence intervals were calculated using multivariable logistic regression. All data were adjusted for sex, age group, smoking status, BMI status and alcohol intake status.

## Results

### Population characteristics

Overall, 1221 patients were registered at the practice during data collection (Table 1). Males comprised the majority of the population (84.9%) and the population had a mean age of 39.7 ± 12.2 years and a mean time registered with the practice of 3.9 ± 4.3 years. Asylum seekers accounted for 14.5% of the patients. Based on their current address, 86 (7.0%) of patients were recorded has having no fixed abode, 152 (12.4%) in a hostel, 167 (13.7%) in a hotel, 88 (7.2%) in supported accommodation and the remaining 645 (52.8%) had a residential address recorded that represented some form of temporary accommodation (permanent living situations do not remain at the specialist practice). Of the total population, 319 (26.1%) were recorded as rough sleeping at some point.

**Table 1:** Characteristics and differences between those with a non-fatal overdose and those without

Characteristic	Total participants (n = 1221)	At least one NFOD on record			*p*-value
		Yes (n = 194)		No (n = 1027)	
	n (% total)	n (% those with overdose)		n (% those without overdose)	
*Age groups (years)*					
16–25	153 (12.5%)	4 (2.1%)		149 (14.5%)	< 0.001
26–35	334 (27.3%)	37 (19.1%)		297 (28.9%)	
36–45	373 (30.5%)	94 (48.4%)		279 (27.2%)	
45 +	361 (29.6%)	59 (30.4%)		302 (29.4%)	
Sex (male)	1037 (84.9%)	154 (79.4%)		883 (86.0%)	0.018
*Body mass index (kg/m*^*2*^*)*					
Normal (18.5–25.0 kg/m^2^)	516 (42.3%)	102 (52.6%)		414 (40.3%)	< 0.001
Underweight (< 18.5 kg/m^2^)	59 (4.8%)	13 (6.7%)		46 (4.5%)	
Overweight/obese (> 25 kg/m^2^)	364 (29.8%)	65 (33.5%)		299 (29.1%)	
Not recorded	281 (23.0%)	14 (7.2%)		267 (26.0%)	
*Ethnicity*					
White	285 (23.3%)	75 (38.7%)		210 (20.4%)	< 0.001
Black/African/Caribbean	139 (11.4%)	11 (5.7%)		128 (12.5%)	
Asian	127 (10.4%)	10 (5.1%)		117 (11.4%)	
Mixed	141 (11.5%)	45 (23.2%)		96 (9.3%)	
Others	44 (3.6%)	0		44 (4.3%)	
Unknown/not recorded	485 (39.7%)	53 (27.3%)		432 (42.1%)	
Asylum seekers	177 (14.5%)	20 (10.3%)		157 (15.3%)	0.071
*Accommodation (from address)*					
No fixed abode	86 (7.0%)	20 (10.3%)		66 (6.3%)	
Hostel	152 (12.4%)	26 (13.4%)		126 (12.3%)	0.019
Hotel	167 (13.7%)	33 (17.0%)		134 (13.0%)	
Supported accommodation	88 (7.2%)	11 (5.7%)		77 (7.5%)	
Unknown/temporary housing	570 (46.7%)	70 (36.1%)		500 (48.7%)	
Rough sleeping (ever recorded)	319 (26.1%)	55 (28.4%)		264 (25.7%)	0.442
*Smoking*					
Never smoker	209 (17.1%)	6 (3.1%)		203 (19.8%)	< 0.001
Ex-smoker	52 (4.3%)	6 (3.1%)		46 (4.5%)	
Current smoker	680 (55.7%)	171 (88.1%)		509 (49.6%)	
No information recorded	280 (22.9%)	11 (5.7%)		269 (26.2%)	
*Cigarettes consumption*					
No. of cigarettes per day (n = 396)	11.3 (8.5%)	12.7 (8.7%)		10.9 (8.4%)	0.032
Grams of tobacco per week (n = 460)	17.5 (22.3%)	32.4 (22.8%)		15.2 (21.4%)	< 0.001
*Alcohol consumption*					
No (no record of it)	681 (55.8%)	59 (30.4%)		622 (60.6%)	< 0.001
Yes	540 (44.2%)	135 (69.6%)		405 (39.4%)	
Alcohol dependent	265 (21.7%)	90 (46.4%)		175 (17.03%)	< 0.001
Not alcohol dependent	275 (50.9%)	45 (33.3%)		230 (56.8%)	
*Alcohol consumption (units/week)*					
Up to 14	196 (48.9%)	38 (40.4%)		158 (51.5%)	0.061
More than 14	205 (51.1%)	56 (59.6%)		149 (48.5%)	
*Alcohol-related information*					
Refused to discuss drinking	19 (1.6%)	4 (2.1%)		15 (1.5%)	0.535
Alcohol overdose	63 (5.2%)	32 (16.5%)		31 (3.0%)	< 0.001
Alcohol/drug referral accepted	59 (4.8%)	20 (10.3%)		39 (3.8%)	< 0.001
Alcohol /drug referral declined	17 (1.4%)	6 (3.1%)		11 (1.1%)	0.028
Education about alcohol provided	294 (24.1%)	80 (41.2%)		214 (20.8%)	< 0.001
*Blood pressure measurement (too few with low BP to include)*					
Normal (90–140 SBP)	810 (67.7%)	153 (80.5%)		657 (65.2%)	< 0.001
High (> 140 SBP)	131 (10.9%)	24 (12.6%)		107 (10.6%)	
No measurement	255 (21.3%)	13 (6.8%)		242 (24.0%)	
*Illicit substance use*					
Substance misuse present	442 (36.2%)	146 (75.3%)		296 (28.8%)	< 0.001
Poly-substance misuse	272 (22.3%)	102 (52.6%)		170 (16.5%)	< 0.001
Cannabis	114 (9.3%)	37 (19.1%)		77 (7.5%)	< 0.001
Cocaine	194 (15.9%)	72 (37.1%)		122 (11.9%)	< 0.001
Heroin	257 (21.0%)	91 (46.9%)		166 (16.2%)	< 0.001
Methadone (misuse of)	22 (1.8%)	10 (5.1%)		12 (1.2%)	< 0.001
Opioid (not otherwise specified)	187 (15.3%)	73 (37.6%)		114 (11.1%)	< 0.001
Synthetic cannabinoids (e.g. mamba, spice)	77 (6.3%)	28 (14.4%)		49 (4.8%)	< 0.001
Benzodiazepine	32 (2.6%)	21 (10.8%)		11 (1.1%)	< 0.001
Ecstasy (MDMA)	6 (0.5%)	4 (2.1%)		2 (0.2%)	0.001
Amphetamines	17 (1.4%)	10 (5.1%)		7 (0.7%)	< 0.001
*Physical health co-morbidities*					
At least one present	462 (37.8%)	129 (66.5%)		333 (32.4%)	–
Respiratory	145 (11.9%)	51 (26.3%)		94 (9.1%)	< 0.001
Asthma	131 (10.7%)	48 (24.7%)		83 (8.1%)	< 0.001
COPD	29 (2.4%)	10 (5.1%)		19 (1.8%)	0.006
BBV	167 (13.7%)	64 (33.0%)		103 (10.0%)	< 0.001
Hepatitis C	157 (12.9%)	62 (32.0%)		95 (9.2%)	< 0.001
HIV	14 (1.1%)	3 (1.5%)		11 (1.1%)	0.568
Cardiovascular	16 (1.3%)	3 (1.5%)		13 (1.3%)	0.753
GI bleed or ulcer	74 (6.1%)	23 (11.9%)		51 (5.0%)	< 0.001
Diabetes type 2	44 (3.6%)	4 (2.1%)		40 (3.9%)	0.209
Epilepsy	35 (2.9%)	15 (7.7%)		20 (1.9%)	< 0.001
Hypertension	71 (5.8%)	11 (5.7%)		60 (5.8%)	0.925
Leg/foot ulcer	51 (4.2%)	15 (7.7%)		36 (3.5%)	0.007
STI	15 (1.2%)	5 (2.6%)		10 (1.0%)	0.063
Migraine	44 (3.6%)	14 (7.2%)		30 (2.9%)	0.003
*Mental health co-morbidities*					
At least one present	619 (50.7%)	164 (84.5%)		455 (44.3%)	–
ADHD	21 (1.7%)	8 (4.1%)		13 (1.3%)	0.005
Depression	410 (33.6%)	112 (57.7%)		298 (29.0%)	< 0.001
Anxiety	276 (22.6%)	90 (46.4%)		186 (18.1%)	< 0.001
PTSD	62 (5.1%)	16 (8.2%)		46 (4.5%)	0.028
Bipolar	21 (1.7%)	7 (3.6%)		14 (1.4%)	0.027
Personality disorder	82 (6.7%)	48 (24.7%)		34 (3.3%)	< 0.001
Psychosis (inc. Schizophrenia)	85 (7.0%)	23 (11.9%)		62 (6.0%)	0.003
Substance misuse	442 (36.2%)	146 (75.3%)		296 (28.8%)	< 0.001
Drug-related mental behaviour	55 (4.5%)	16 (8.2%)		39 (3.8%)	0.006
Alcohol related	20 (1.6%)	9 (4.6%)		11 (1.1%)	< 0.001
Recorded self-harm or suicide attempt	185 (15.1%)	81 (41.7%)		104 (10.1%)	< 0.001

Values are mean (± SD) or n (%) where % is the percentage within the category and p values are testing the heterogeneity between the baseline characteristics

COPD, Chronic obstructive pulmonary disease; BBV, Blood borne virus; HIV, Human Immunodeficiency virus; GI, gastrointestinal; STI, Sexually transmitted infection; ADHD, Attentional deficit hyperactivity disorder; PTSD, Post-traumatic stress disorder; Drug-related mental behaviour, mental health conditions related to drug use

A total of 462 (37.8%) had at least one chronic condition reported. Of the conditions included, blood-borne viruses (BBV) were recorded in 167 (13.7%) of the population, and respiratory conditions were recorded in 145 (11.9%). There was a high prevalence of mental health conditions within the population, with 619 (50.7%) having at least one mental health condition documented. Depression was the most common (n = 410, 33.6%), followed by anxiety (n = 276, 22.6%). An attempt at suicide or self-harm at any time had been recorded in 185 (15.2%).

### Substance use

Current smokers made up 55.7% (n = 680) of the total patient group, and 44.2% (n = 540) were recorded as consuming alcohol. This included 265 (21.7%) noted to be problem drinkers.

**Fig. 1:**
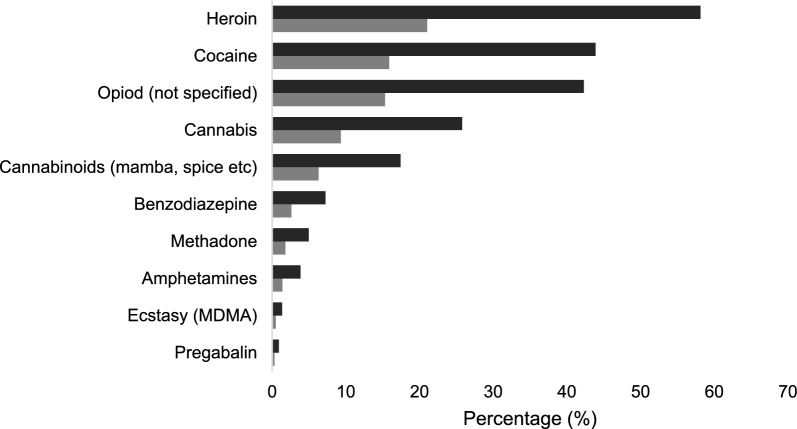
Substance of use in all patients with coded substance misuse (n = 442). Percentages represent that of the substance misuse population, n = 442 (dark grey) and the total population, n = 1221 (light grey). Methadone refers to the misuse of methadone, not prescribed use

Illegal substance misuse was identified in 442 patients (36.2%). Heroin was the most common substance of use, with 257 (58.1%) of the substance misuse population reported as users (21.0% of the total population). As shown in Fig. 1, other common substances included cocaine (194, 43.9% substance misuse population; 15.9% total population), cannabis (25.8% of substance misuse population, 9.3% total population) and synthetic cannabinoids such as mamba and spice (n = 77, 17.4% substance misuse, 6.3% total population).

### Non-fatal overdoses

In total, 194 patients (15.9%) were recorded as ever having had a NFOD, with 428 overdoses between them. The median number of overdoses was 1, with 100 patients (51.5% of patients with NFOD) having just one recorded episode. Two overdoses were recorded in 45 people (23.2% of patients with NFOD), three in 22 people (11.3% of patients with NFOD), four in 12 people (6.2% of patients with NFOD) and 15 people (7.6% of patients with NFOD) had 5 or more overdoses recorded. The maximum number of episodes recorded for a single patient was 21. In the five years prior to the data collection, 94 patients (7.7% of all patients) had a recorded overdose.

No information beyond the coded term of substance overdose was available for 121 (28.2%) of the 428 overdoses. In total, there were 264 (61.7%) incidents that had a recorded substance of overdose, and 208 (48.6%) had information regarding the management.

### Substance of overdose

Of the 264 NFODs that had a substance recorded, single substance overdoses were seen in exactly half (50.0%) of these, with poly-substance overdoses accounting for the other half. Alcohol use was implicated in a further 51 (19.3%) of these overdoses.

**Fig. 2:**
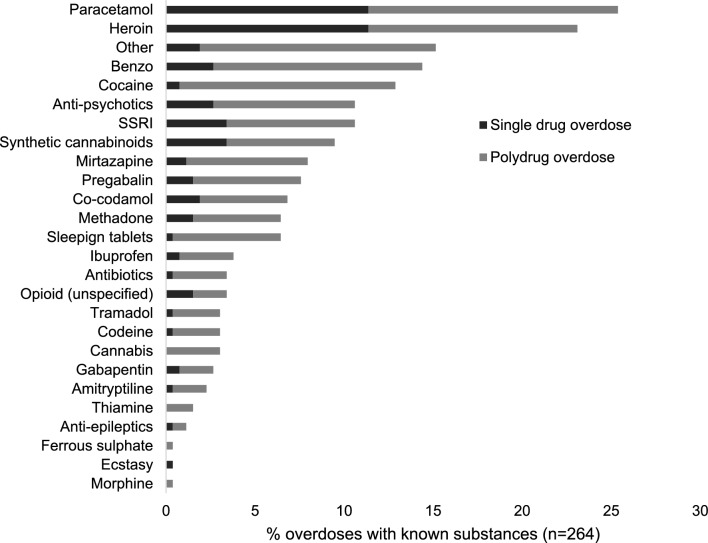
Substance of overdose for 264 overdoses of known substances. (Full bar length = overall total, broken down for polydrug overdoses (dark grey) and single drug overdoses (light grey))

As seen in Fig. 2, for single substance overdoses (n = 132), the most commonly identified substances were: heroin (n = 30, 22.7%), paracetamol (n = 30, 22.7%), synthetic cannabinoids (n = 9, 6.8%), SSRIs (n = 9 6.8%), anti-psychotics (n = 7, 5.3%), benzodiazepines (n = 7, 5.3%) and co-codamol (n = 5, 3.8%). For polysubstance overdoses, paracetamol (n = 37, 28.0%) was the most common substance followed by heroin (n = 31, 23.5%), cocaine (n = 32, 24.2%), benzodiazepines (n = 31, 23.5%), anti-psychotics (n = 21, 15.9%), SSRIs (n = 19, 14.4%), mirtazapine (n = 18, 13.6%), synthetic cannabinoids (n = 16, 12.1%), pregabalin (n = 16, 12.1%), sleeping tablets – unspecified, but could include benzodiazepines (n = 16, 12.1%), co-codamol (n = 13, 9.8%) and methadone (n = 13, 9.8%).

Of the 194 who had a recorded overdose, 146 (75.3%) had coded substance abuse on record. Heroin was the most common substance of use, with 91 (46.9% NFOD population) reported to be users. Other opioids not specified (n = 74, 37.6%), cocaine (n = 54, 37.1%), cannabis (n = 37, 19.1%), synthetic cannabinoids (n = 28, 14.4%) and benzodiazepines (n = 22, 11.3%) were also popular substances of abuse.

### Use of emergency services in NFOD

Of the 208 overdoses with management information, 110 (52.9%) had an ambulance call out (Table 2). Of these, 21 (10.1%) involved the patient self-discharging from the ambulance prior to reaching the hospital. Naloxone was administered in 32 (15.4%) cases, primarily given by the ambulance service. Overall, 148 (71.2%) NFODs led to a presentation to the ED, with self-discharge before assessment in 21 cases (10.1%).

**Table 2:** Services used for non-fatal overdoses (total = 428 overdoses)

Management	n	% total overdoses	% overdoses with recorded information
No management information	220	51.4	–
Ambulance called	110	25.7	52.9
ED attended	148	34.6	71.2
Ambulance + ED	78	18.2	37.5
Ambulance or ED attended	181	42.3	87.0
Naloxone administered	32	7.5	15.4
Hospital admission	57	13.3	27.4
Self-discharged from ambulance	21	4.9	10.1
Self-discharged from hospital/ED	21	4.9	10.1

In total, 57 overdose events (27.4%) led to hospital admissions. For 25 of these 57, a length of stay was recorded. The majority (n = 14) stayed for one day, 7 for two days, two individuals for 3 days, one person for 4 days, one for 11 days and one for 25 days. The short single-day stays were reported for observation, whereas longer stays were due to co-existing issues, including aspiration pneumonia (25 days), mental health reasons (11 days) and leg ulcers (3 days).

### Circumstances leading to NFOD

In some of the medical notes, there was descriptive text regarding the overdose. In general, two subsets of overdoses appeared. Firstly, was the group that intended to self-harm or attempt suicide. In 50 cases (12% of NFODs with available data), direct references to mental health were made, with 25 (9% of NFODs with available data) noting suicidal intent. These were often triggered by life events such as relationship breakups or a family member dying. These overdoses often involved taking multiple different street substances and/or prescribed medications.

A second subset (n = 91, 44% of NFOD with available data) involved regular substance users who overdosed on street drugs. This group appeared more likely to self-discharge from emergency care. Of 91 overdoses identified as caused by street drugs (predominantly heroin and cocaine), self-discharge from ambulances or ED occurred in 31 (34.1%) cases accounting for 73.8% of the self-discharge events. While it is not possible to be certain, many overdose events were likely unintentional due to factors such as reduced tolerance from being in prison noted.

### Risk factors for non-fatal overdose

Several factors were associated with individuals with a recorded NFOD compared to those without (Table 3). Male sex was associated with a reduced risk of NFOD (OR: 0.52, 95% CI 0.33–0.81), white ethnicity a greater risk compared to Black/African/Caribbean (OR: 0.42, 95% CI 0.21–0.85) or Asian (OR: 0.42, 95% CI 0.20–0.89) and age 36–45 associated with higher risks (OR: 3.86, 95% CI 1.33–11.22).

**Table 3:** Factors associated with non-fatal overdose

Characteristic	n (% total participants)	Odds ratio (OR)	95% CI
*Age (years)*			
16–25	153 (12.5%)	Ref	
26–35	334 (27.3%)	2.02	0.68–6.05
36–45	**373 (30.5%)**	**3.86**	**1.33**–**11.22**
45+	361 (29.6%)	1.97	0.66–5.82
*Sex*			
Female	184 (15.1%)	Ref	
Male	1037 (84.9%)	0.52	0.33–0.81
*Body mass index (kg/m*^*2*^*)*			
Normal (18.5–25.0 kg/m^2^)	516 (42.3%)	Ref	
Underweight (< 18.5 kg/m^2^)	59 (4.8%)	1.01	0.50–2.05
Overweight/obese (> 25 kg/m^2^)	364 (29.8%)	1.14	0.78–1.67
Not recorded	281 (23.0%)	0.76	0.37–1.55
*Ethnicity*			
White	285 (23.3%)	Ref	
Black/African/Caribbean	**139 (11.4%)**	**0.42**	**0.21**–**0.85**
Asian	**127 (10.4%)**	**0.42**	**0.20**–**0.89**
Mixed	141 (11.5%)	1.36	0.85–2.17
Unknown/not recorded	485 (39.7%)	0.68	0.44–1.06
*Number of years registered at practice*			
less than one year	434 (35.5%)	Ref	
1–2 years	207 (16.9%)	0.94	0.54–1.65
2–4 years	183 (15.0%)	0.7	0.38–1.27
4–6 years	134 (11.0%)	0.72	0.40–1.30
> 6 years	263 (21.5%)	0.71	0.42–1.20
*Accommodation from address*			
Supported accommodation	88 (7.2%)	Ref	
No fixed abode	86 (7.0%)	1.12	0.60–2.09
Hostel	152 (12.4%)	0.72	0.42–1.24
Hotel	167 (13.7%)	1.10	0.65–1.85
Unknown/temporary housing	570 (46.7%)	0.80	0.38–1.68
Rough sleeping (ever recorded)	319 (26.1%)	1.12	0.76–1.64
*Smoking*			
Never smoker	209 (17.1%)	Ref	
Ex-smoker	**52 (4.3%)**	**5.45**	**1.41**–**21.00**
Current smoker	**680 (55.7%)**	**10.84**	**3.84**–**30.58**
No information recorded	280 (22.9%)	2.79	0.79–9.82
*Alcohol consumption*			
Never	681 (55.8%)	Ref	
Ever drinker	**540 (44.2%)**	**2.19**	**1.51**–**3.17**
*Alcohol dependence*			
Alcohol dependent	265 (49.1%)	Ref	
Not alcohol dependent	**275 (50.9%)**	**2.28**	**1.59**–**3.29**
*Alcohol consumption (units/week)*			
Up to 14	196 (48.9%)	Ref	
More than 14	205 (51.1%)	1.18	0.71–1.99
*Alcohol related complications*			
Delirium tremens/withdrawal	**19 (1.56%)**	**4.1**	**1.39**–**12.12**
*Blood pressure measurement*			
Normal (90–140 SBP)	810 (67.7%)	Ref	
High (> 140 SBP)	131 (10.9%)	0.93	0.54–1.57
No measurement	255 (21.3%)	1.12	0.49–2.58
*Illicit substance use*			
Any illicit substance misuse	**442 (36.2%)**	**5.86**	**3.58**–**9.58**
Poly-substance misuse	**272 (22.3%)**	**2.73**	**1.88**–**3.95**
Cannabis	**114 (9.3%)**	**1.77**	**1.11**–**2.83**
Cocaine	**194 (15.9%)**	**2.35**	**1.60**–**3.47**
Heroin	**257 (21.0%)**	**2.14**	**1.48**–**3.12**
Opioid (not specified)	**187 (15.3%)**	**2.19**	**1.47**–**3.26**
Synthetic cannabinoids (mamba, spice etc.)	**77 (6.3%)**	**1.74**	**1.01**–**2.99**
Benzodiazepine	**32 (2.6%)**	**5.45**	**2.41**–**12.31**
Ecstasy (MDMA)	**6 (0.5%)**	**6.72**	**1.15**–**39.20**
Amphetamines	**17 (1.4%)**	**4.81**	**1.62**–**14.24**
*Physical co-morbidities*			
Respiratory	**145 (11.9%)**	**1.84**	**1.20**–**2.81**
Asthma	**131 (10.7%)**	**1.96**	**1.26**–**3.04**
COPD	29 (2.4%)	1.63	0.69–3.85
BBV	**167 (13.7%)**	**2.19**	**1.46**–**3.28**
Hepatitis C	**157 (12.9%)**	**2.25**	**1.49**–**3.39**
HIV	14 (1.1%)	0.71	0.18–2.73
Cardiovascular	16 (1.3%)	1.19	0.30–4.64
GI bleed or ulcer	74 (6.1%)	1.51	0.85–2.68
Type 2 diabetes	44 (3.6%)	0.38	0.12–1.13
Hypertension	71 (5.8%)	0.75	0.36–1.56
Leg/foot ulcer	51 (4.2%)	1.15	0.59–2.24
STI	15 (1.2%)	1.08	0.33–3.50
Migraine	**44 (3.6%)**	**2.21**	**1.06**–**4.62**
*Mental health co-morbidities*			
ADHD	**21 (1.7%)**	**3.12**	**1.14**–**8.52**
Depression	**410 (33.6%)**	**1.79**	**1.26**–**2.55**
Anxiety	**276 (22.6%)**	**2.27**	**1.59**–**3.26**
PTSD	62 (5.1%)	1.75	0.90–3.42
Bipolar	21 (1.7%)	1.14	0.43–3.03
Personality disorder	**82 (6.7%)**	**4.92**	**2.96**–**8.17**
Psychosis (inc Schizophrenia) disorder	85 (7.0%)	1.38	0.80–2.38
Drug-related mental and behaviour	55 (4.5%)	1.38	0.73–2.63
Alcohol related	**20 (1.6%)**	**3.69**	**1.28**–**10.61**
Recorded self-harm or suicide attempt	**185 (15.1%)**	**3.53**	**2.38**–**5.22**

Bold numbers indicate significant risk factors

COPD, Chronic obstructive pulmonary disease; BBV, Blood borne virus; HIV, Human Immunodeficiency virus; GI, gastrointestinal; STI, Sexually transmitted infection; ADHD, Attentional deficit hyperactivity disorder; PTSD, Post-traumatic stress disorder

Being an ex or current smoker increased the odds of a NFOD by 5.45 (95% CI 1.41–21.00) and 10.84 (95% CI 3.84–30.58) times, respectively, as did being ever reported as an alcohol drinker (OR: 2.19, 95% CI 1.51–3.17). However, being non-alcohol dependent increased the likelihood of NFOD compared to a reported alcohol dependence (OR: 2.28, 95% CI 1.59–3.29).

Having any illicit substance use substantially increased the odds of having a NFOD (OR: 5.86, 95% CI 3.58–9.58). Records of cannabis (OR: 1.77, 95% CI 1.11–2.83), cocaine (OR: 2.35 95% CI 1.60–3.47), heroin (OR: 2.14, 95% CI 1.48–3.12), opioids not specified (OR: 2.19 95% CI 1.47–3.26) and synthetic cannabinoids (OR: 1.74, 95% CI 1.01–2.99) were positively associated with NFOs.

In general, physical or mental health conditions increased the risk of an NFO, including the presence of a respiratory condition (OR: 1.84, 95% CI 1.20–2.81), particularly asthma (OR: 1.96, 95% CI 1.26–3.04), or a blood-borne virus (OR: 2.19, 95% CI 1.46–3.28) or known migraines (OR: 2.21, 95% CI 1.06–4.62). Further risks included having diagnosed anxiety (OR: 2.27, 95% CI 1.59–3.26), depression (OR: 1.79, 95% CI 1.26–2.55), ADHD (OR: 3.12, 95% CI 1.14–8.52) or a personality disorder (OR: 4.92, 95% CI 2.96–8.17).

## Discussion

### Summary of key findings

This study demonstrates a high proportion (15.9%) of PEH registered at a specialist primary care centre had a recorded NFOD. Risk factors identified for these overdoses included an increased risk in women, those of white ethnicity and those aged 36–45, the use of tobacco and alcohol, having a known substance misuse disorder and having at least one chronic health condition, specifically respiratory, blood borne viruses, migraines, anxiety and depression. The variety of substances used to overdose combined with the qualitative information suggests two types of overdoses: accidental overdose in street substance users and self-harm attempts. These overdoses frequently required help from emergency services, from which a significant number of patients self-discharged.

### Interpretation

The two types of overdose identified, accidental overdose and overdose with suicidal or self-harm intent, are not often considered concurrently. However, both are large contributors to fatality amongst PEH. In England and Wales in 2021, 35% of deaths in PEH were caused by substance overdoses and 13.4% by suicide [2]. These two types of overdose are more likely to be at ends of a spectrum with considerable overlap rather than being distinct classes, with suicidal thoughts shown to be present in almost 50% of illicit NFOD [17] and suicidal ideation a risk factor for accidental overdose [18].

The mixture of illicit, over the counter, and prescription drugs reported in the NFOD most likely reflect the combination of these two types of overdoses, with paracetamol most commonly used for self-harm attempts in PEH [19], and heroin, benzodiazepines and cocaine frequent in street drug overdoses [10]. The importance of considering the type of overdose lies in the preventative approach from a health care perspective. Although sharing similarities that may benefit from comparable holistic and mental health care, there may be some variation in the approach to dealing with patients in each group. For example, ensuring naloxone for those likely to overdose on heroin vs limiting the prescribing of medicines potentially associated with overdose. Alternative approaches are in place in other UK cities with opportunities for heroin assisted treatment and supervised drug consumption in Glasgow, for example.

Alongside factors previously identified to be associated with NFOD including being female [9], a polysubstance user [9], being white [20] and known substance misuse, the most novel risk factors identified related to the association with chronic health conditions. This is already recognised from a mental health perspective, with mental health disorders shown to be the second highest risk factor for overdose behind substance use in a meta-analysis [21]. Chronic physical health conditions have not been previously considered a risk factor, but we do know there is a high level of multi-morbidity within this population, with a mean of 2.2 mental health problems and 5.4 physical health problems per person reported in a group of PEH with a recent NFOD [10].

Respiratory disease (particularly asthma), BBV (particularly Hep C) and migraines were associated with an increased risk of NFOD. While the timing of diagnosis and overdose events remains unknown, thus making the direction of these relationships unclear, chronic health conditions come with a large burden to PEH physically, mentally and socially [22].

Most overdose incidents reported here (87%) had contact with emergency services (ambulance or the emergency department), although this is likely an overestimation if considering all episodes of overdose in the community as many overdoses are likely to unrecorded where no contact with health services were made. Just over half (53%) arrived by ambulance. Self-discharge from emergency services was high, with 14% of those that attended ED self-discharging and 19% of those that had an ambulance call out refusing help. Similar reports of self-discharge from the ED have been documented [11]. Self-discharge following a poisoning episode could be associated with an increased risk of death [23]. Waiting environment, the length of time of the wait and waiting around other people in the emergency department could contribute to the self-discharge before assessment [24].

### Strengths and weaknesses

This study is novel considering existing literature focus mainly on fatal overdose. However there are some limitations. While a large number of patient data and non-fatal overdose events were analysed, data originated from one specialist homelessness general practice and this can have limited generalisability. The recorded NFOD here only captures those that had medical attention, but it is likely a number of NFOD happen without medical attention in the community and in the streets. The EMIS system used to identify NFOD only allowed searching of coded instances of NFOD, meaning some that were not coded were missed from the evaluation. Furthermore, the free text data in discharge letters was analysed by only one researcher (a final year graduate medical student with a PhD degree), thus the reliability of this was not assessed. There were also missing data on the circumstances surrounding the overdoses. Finally, the population included at the practice also includes asylum seekers (14%) and it is worth noting that this population will vary in terms of their health care needs and less is known about substance use patterns in this population.

### Further research

Future research should identify the direction and temporal association between physical and mental health conditions and overdose as well as consider the relationship between treatment burden and overdose. A greater understanding surrounding the psychopathology of mental health conditions in PEH such as anxiety and depression could help target primary prevention strategies while extending into more acute stressors could also improve our understanding around timings of overdose. Overall prevention strategies need to consider the type of any previous overdose, if present, and target those with risk factors for NFOD, inclusive of chronic mental and physical health conditions. From a medication perspective, this should focus on promoting user understanding around risks related to overdose from both prescribed and illicit substances. Language, health literacy and cultural barriers can often hinder effective communications between healthcare professionals and PEH leaving people at risk of medication misadventures and overdose due to lack of adequate knowledge and understanding. Future intervention should focus addressing these factors on an individual basis, but more substantially from a population perspective, such as the use of social media to target younger PEH, particularly in relation to novel psychoactive substance and their risks [25]. Given that NFOD are associated with future overdose mortality [8], research and practice based on multisector collaboration is key to document and apply prevention approaches to NFOD wherever identified including in the community, streets, temporary accommodations. When considering prevention approaches, it is important to consider wider determinants of health including barriers of PEH access to primary care such as experiences of stigma and discrimination in public health and healthcare setting, exclusion due to lack of proof of address, and perceived fragmentation of services [26–30].

## Conclusion

Female sex, substance use disorder (legal and illegal substance) and chronic mental and physical comorbidities increased the odds of a NFOD in PEH. Identifying people with NFOD and awareness of risk factors can enable primary care providers to apply prevention actions such as provision of overdose awareness and naloxone to avoid future fatal overdose events. Future work should explore the role of chronic physical conditions and their treatment on NFOD risk.

## Supplementary Information

Below is the link to the electronic supplementary material.

Supplementary file1 (DOCX 29 KB)

## References

[1] Office for National Statistics. Deaths related to drug poisoning in England and Wales: 2021 registrations. Health Stat. Q. 2022. Available from: https://www.ons.gov.uk/peoplepopulationandcommunity/birthsdeathsandmarriages/deaths/bulletins/deathsrelatedtodrugpoisoninginenglandandwales/2021registrations. Accessed 14 Sep 2024.

[2] Office for National Statistics. Deaths of homeless people in England and Wales: 2021 registrations. 2022 [cited 2022 Dec 12]. Available from: https://www.ons.gov.uk/peoplepopulationandcommunity/birthsdeathsandmarriages/deaths/bulletins/deathsofhomelesspeopleinenglandandwales/2021registrations#regional-analysis. Accessed 14 Sep 2024.

[3] Watts B, Bramley G, Pawson H, et al. The homelessness monitor: England 2022. Available from: https://www.crisis.org.uk/media/246994/the-homelessness-monitor-england-2022_report.pdf. Accessed 14 Sep 2024.

[4] Brown RT, Kiely DK, Bharel M, et al. Geriatric syndromes in older homeless adults. J Gen Intern Med. 2012;27:16–22. 10.1007/s11606-011-1848-9. 21879368 10.1007/s11606-011-1848-9 PMC3250555

[5] Field H, Hudson B, Hewett N, et al. Secondary care usage and characteristics of hospital inpatients referred to a UK homeless health team: a retrospective service evaluation. BMC Health Serv Res. 2019;19:1–15. 10.1186/s12245-023-00526-9. 31752857 10.1186/s12913-019-4620-1 PMC6868755

[6] Bowen M, Marshall T, Yahyouche A, et al. Multimorbidity and emergency department visits by a homeless population: a database study in specialist general practice. Br J Gen Pract. 2019;69:e515–25. 10.3399/bjgp19X704609. 31262848 10.3399/bjgp19X704609 PMC6607834

[7] National Institute for Health and Care Excellence. Integrated health and social care for people experiencing homelessness—NICE Guidelines (NG214). NICE Guidelines. 2022. Available from: https://www.nice.org.uk/guidance/ng214. Accessed on 14 Sep 2024. 35442601

[8] Caudarella A, Dong H, Milloy MJ, et al. Non-fatal overdose as a risk factor for subsequent fatal overdose among people who inject drugs. Drug Alcohol Depend. 2016;162:51–5. 10.1016/j.drugalcdep.2016.02.024. 26993373 10.1016/j.drugalcdep.2016.02.024 PMC4833586

[9] Armoon B, Bayani A, Griffiths MD, et al. Prevalence and high-risk behaviors associated with non-fatal overdose among people who use illicit opioids: a systematic review and meta-analysis. J Subst Use. 2022;27:569–84. 10.1080/14659891.2021.1978112.

[10] Lowrie R, McPherson A, Mair FS, et al. Baseline characteristics of people experiencing homelessness with a recent drug overdose in the PHOENIx pilot randomised controlled trial. Harm Reduct J. 2023;20:1–18. 10.1186/s12954-023-00771-4. 37016418 10.1186/s12954-023-00771-4 PMC10071267

[11] Paudyal V, Ghani A, Shafi T, et al. Clinical characteristics, attendance outcomes and deaths of homeless persons in the emergency department: implications for primary health care and community prevention programmes. Public Health. 2021;196:117–23. 10.1016/j.puhe.2021.05.007. 34182257 10.1016/j.puhe.2021.05.007

[12] Paudyal V, Vohra N, Price M, et al. Key causes and long-term trends related to emergency department and inpatient hospital admissions of homeless persons in England. Int J Emerg Med. 2023;16:1–13. 10.1186/s12245-023-00526-9. 37550625 10.1186/s12245-023-00526-9 PMC10405435

[13] Vohra N, Paudyal V, Price MJ. Homelessness and the use of emergency department as a source of healthcare : a systematic review. Int J Emerg Med. 2022;15:32. 10.1186/s12245-022-00435-3. 35902803 10.1186/s12245-022-00435-3 PMC9330962

[14] National records of Scotland. Homeless Deaths 2022. Available from: https://www.nrscotland.gov.uk/statistics-and-data/statistics/statistics-by-theme/vital-events/deaths/homeless-deaths/2022. Accessed 05 Sep 2024.

[15] The Scottish Goverment. Drug Deaths Taskforce Response: A Cross Government Approach. 2023. Available from: https://www.gov.scot/publications/drug-deaths-taskforce-response-cross-government-approach. Accessed 05 Sep 2024.

[16] Drug Research Network Scotland. Drug Research Network Scotland (DRNS). 2017. Available from: https://drns.ac.uk/. Accessed 05 Sep 2024.

[17] Neale J. Suicidal intent in non-fatal illicit drug overdose. Addiction. 2000;95:85–93. 10.1046/j.1360-0443.2000.951859.x. 10723833 10.1046/j.1360-0443.2000.951859.x

[18] Richer I, Bertrand K, Vandermeerschen J, et al. A prospective cohort study of non-fatal accidental overdose among street youth: the link with suicidal ideation. Drug Alcohol Rev. 2013;32:398–404. 10.1111/dar.12003. 23130603 10.1111/dar.12003

[19] Clements C, Farooq B, Hawton K, et al. Self-harm in people experiencing homelessness: investigation of incidence, characteristics and outcomes using data from the multicentre study of self-harm in England. BJ Psych Open. 2022;8:1–8. 10.1192/bjo.2022.30. 10.1192/bjo.2022.30 PMC9059614 35317881

[20] Riggs KR, Hoge AE, DeRussy AJ, et al. Provision and accessibility of primary healthcare services for people who are homeless: a qualitative study of patient perspectives in the UK. Br J Gen Pract. 2022;12:E526–36. 10.3399/bjgp19X704633. 10.3399/bjgp19X704633 PMC6650120 31307999

[21] Brady JE, Giglio R, Keyes KM, et al. Risk markers for fatal and non-fatal prescription drug overdose: a meta-analysis. Inj Epidemiol. 2017;4:1–24. 10.1186/s40621-017-0118-7. 28762157 10.1186/s40621-017-0118-7 PMC5545182

[22] Jones C, Mair FS, Williamson AE, et al. What is the treatment burden for people experiencing homelessness with a recent non-fatal overdose? BJGP. 2023;73(735):e728–34. 10.3399/BJGP.2022.0587. 37429734 10.3399/BJGP.2022.0587 PMC10355813

[23] Vallersnes OM, Jacobsen D, Ekeberg Ø, et al. Mortality and repeated poisoning after self-discharge during treatment for acute poisoning by substances of abuse: a prospective observational cohort study. BMC Emerg Med. 2019;19:1–7. 10.1186/s12873-018-0219-9. 30634924 10.1186/s12873-018-0219-9 PMC6329053

[24] Currie J, Stafford A, Hutton J, et al. Optimising access to healthcare for patients experiencing homelessness in hospital emergency departments. Int J Environ Res Public Health. 2023;20(3):2424. 10.3390/ijerph20032424. 36767794 10.3390/ijerph20032424 PMC9916150

[25] Romer D, Moreno M. Digital media and risks for adolescent substance abuse and problematic gambling. Pediatrics. 2017;140:S102–6. 10.1542/peds.2016-1758L. 29093042 10.1542/peds.2016-1758L PMC5658796

[26] Kaur S, Parbir M, Paudyal V. Provision of services to persons experiencing homelessness during the COVID-19 pandemic : a qualitative study on the perspectives of homelessness service providers. Health Soc Care Commun. 2021. 10.1111/hsc.13609. 10.1111/hsc.13609 PMC8653035 34668258

[27] Paudyal V, MacLure K, Buchanan C, et al. When you are homeless, you are not thinking about your medication but your food, shelter or heat for the night’: behavioural determinants of the homeless population adherence to prescribed medicines. Public Health. 2017;148:1–8. 10.1016/j.puhe.2017.03.002. 28404527 10.1016/j.puhe.2017.03.002

[28] Kaushal R, Jagpal P, Khanal S, et al. Representation of homeless persons and coding of homelessness in mainstream general practices: a descriptive evaluation using healthcare utilisation data. BJGP Open. 2021. 10.3399/BJGPO.2021.0050. 34045292 10.3399/BJGPO.2021.0050 PMC8450878

[29] Paudyal V, Maclure K, Forbes-McKay K, et al. ’If i die i die i don’t care about my health’ perspectives on self-care of people experiencing homelessness. Heal Soc care commun. 2020;28:160–72. 10.1111/hsc.12850. 10.1111/hsc.12850 31490598

[30] Gunner E, Chandan SK, Yahyouche A, et al. Provision and accessibility of primary healthcare services for people who are homeless: a qualitative study of patient perspectives in the UK. Br J Gen Pract. 2019;69:E526–36. 10.3399/bjgp19X704633. 31307999 10.3399/bjgp19X704633 PMC6650120

