# Supplementary material for: Patterns, circumstances and risk factors associated with non-fatal substance overdose in a cohort of homeless population: an observational study
Source: Int J Clin Pharm. 2024 Nov 19;47(1):107–18. doi: 10.1007/s11096-024-01812-z (PMC11748478; doi:10.1007/s11096-024-01812-z)
Supplement: Supplementary file 1 — Supplementary file1 (DOCX 29 KB) [file 11096_2024_1812_MOESM1_ESM.docx]

**Electronic Supplementary Material 1:** Search terms for non-fatal drug overdose on EMIS Web

| **Parent branch** | **Child branches** | |
| --- | --- | --- |
| [X]Deliberate drug overdose/ other poisoning |  |  |
| Accidental drug overdose |  |  |
| Accidental poisoning by drugs, medicines and biologicals |  |  |
| OD – overdose of drug |  |  |
| Intentional drug overdose |  |  |
| Fentanyl overdose | Accidental fentanyl overdose  Intentional fentanyl overdose |  |
| Heroin overdose | Accidental heroin overdose  Intentional diamorphine overdose |  |
| Benzodiazepine overdose | Accidental overdose of benzodiazepine  Alprazolam overdose  Bromazepam overdose  Chlordiazepoxide overdose  Clonazepam overdose  Clorazepate overdose  Diazepam overdose  Flunitrazepam overdose  Intentional benzodiazepine overdose  Ketazolam overdose  Loprazolam overdose  Lorazepam overdose  Lormetazepam overdose  Medazepam overdose  Midazolam overdose  Nitrazepam overdose  Overdose of temazepam  Oxazepam overdose  Prazenam overdose  Triazolam overdose |  |
| Methadone overdose | Accidental methadone overdose  Intentional methadone overdose |  |
| Gabapentin overdose | Accidental gabapentin overdose  Intentional gabapentin overdose |  |
| Buprenorphine poisoning | Accidental buprenorphine poisoning  Buprenorphine overdose  Intentional buprenorphine poisoning |  |
| Ketamine overdose | Accidental ketamine overdose  Intentional ketamine overdose |  |
| Overdose of cocaine | Accidental overdose by cocaine  Intentional overdose by cocaine  Overdose of crack cocaine |  |
| Poisoning caused by paracetamol | Accidental paracetamol poisoning  Intentional paracetamol poisoning  Paracetamol overdose |  |
| Overdose of codeine | Accidental overdose by codeine  Intentional overdose by codeine |  |
| [X]Intentional self poisoning by exposure to psychotropic drugs |  |  |
| [X]Intentional self poisoning by and exposure to nonopioid analgesics |  |  |
| [X]Intentional self poisoning by and exposure to sedative hypnotics |  |  |
| 5-HT3-receptor antagonist overdose | Accidental 5-HT3-receptor antagonist overdose  Intentional 5-HT3-receptor antagonist overdose |  |
| Barbiturate overdose | Amylobarbitone overdose  Butabarbitone overdose  Cyclobarbitone overdose  Methylphenobarbitone overdose  Phenobarbitone overdose  Primidone overdose  Quinalbarbitone overdose  Thiopental overdose |  |
| Poisoning caused by cannabinoid receptor agonist |  |  |
| Opiate antagonist overdose | Accidental poisoning by opiate antagonists  Opiate antagonist overdose  Poisoning by levallorphran  Poisoning by nalorphine  Poisoning by naloxone |  |
| Overdose of analgesic drug | Buprenorphine overdose  Heroin overdose  Ketamine overdose  Levorphanol overdose  Meptazinol overdose  Methadone analogue overdose  Methadone overdose  Morphine overdose  Nalbuphine overdose  Overdose of codeine  Overdose of dihydrocodeine  Overdose of nonopioid analgesic  Pentazocine overdose  Pethidine analogue overdose  Phenazocine overdose |  |
| Overdose of opiate | Accidental overdose of opiate  Buprenorphine overdose  Heroin overdose  Intentional overdose of opioid receptor antagonist  Meptazinol overdose  Morphinan opioid overdose  Overdose of codeine  Overdose of dihydrocodeine  Pentazocine overdose  Pethidine overdose  Pethidine analogue overdose  Phenazocine overdose |  |
| Psychotropic overdose | Hydroxyzine overdose | Accidental  Intentional |
|  | Neuroleptic overdose | Benperidol overdose  Chlorpromazine overdose  Clozapine overdose  Diphenylbutylpiperidone overdose  Droperidol overdose |
|  | Overdose of antidepressant drug |  |
|  | Overdose of hallucinogenic drug | Cannabis overdose  Ketamine overdose  Mescaline overdose  Overdose of lysergic acid diethylamide  Psilocybin overdose |
|  | Psychostimulant overdose | Overdose of cocaine  Central nervous system stimulant overdose  Amfetamine and/or emfetamine derivative overdose |
|  | Selective serotonin re-uptake inhibitor overdose | Citalopram overdose  Fluoxetine overdose  Fluvoxamine overdose  Nefazodone overdose  Paroxetine overdose  Sertraline overdose  Venlafaxine overdose  Zuclopenthixol decanoate overdose |
|  | Valproate overdose |  |
|  | Sedative overdose | Amylobarbitone overdose  Benzodiazepine overdose  Buspirone overdose  Butabarbitone overdose  Chloral and/ or chloral derivative overdose  Chlormezanone overdose  Cyclobarbitone overdose  Meprobomate overdose  Methylphenobarbitone overdose  Methylprylone overdose  Paraldehyde overdose  Quinalbarbitone overdose  Zolpidem overdose  Zopiclone overdose |
| Overdose | [includes]  Acute overdose  Accidental overdose  Intentional overdose  Opiate antagonist overdose  Overdose of opiate  Psychotropic overdose |  |

**Electronic Supplementary Material 2:** Information recorded for each patient from the practice on EMIS Web

| **Factor** | **Details of term codes** | **Details** |
| --- | --- | --- |
| Patient details |  | Age, gender, DOB, ethnicity, address (to determine accommodation status) |
| Accommodation status | Accommodation status  Homeless  Residence and accommodation circumstances – finding  H/O sleeping rough  History or rough sleeping  Homeless  No longer homeless  Hostel  Lives in supported home | Not sure how well this is completed for everyone, but some information |
| Asylum status | Asylum seeker  Failed asylum seeker | Date, code term |
| Registration history |  | Gives information about how many times registered at this practice |
| Body weight | Body weight | Every date so can document changes |
| BMI | Body mass index |  |
| Blood pressure | O/E blood pressure reading |  |
| Substance of use | See below – search criteria for substance misuse | Date, code term, associated text input, problem status (active, past…) |
| Overdose episodes | See below criteria (table) | Date, code term, associated text (will also go back through files to note down details) |
| Referral to drug services | Referral to drug abuse counsellor  Referral to drug worker  Referral to drug therapist  Referral to drug treatment centre  Referral to community drug dependency team  Referral to community drug and alcohol team  Referral to drug addiction rehabilitation service  Referral to CARAT drug service | Date, code term, associated text |
| Nalxone / buprenoprhine documented | Naloxone  Naloxone Hydrochloride  Drug addiction therapy using buprenorphine and naloxone  Naloxone hydrochloride injection solution ampoule (codes included all dose types) | Date, code term, associated text (this likely only captures some as most given training and naloxone by drug and alcohol team) |
| Alcohol use | See below table for details | Date, code term, associated text, units/week |
| Alcohol related complications | Alcoholic liver damage  Alcoholic cirrhosis of liver  Alcohol withdrawal syndrome (delirium…)  Alcoholic fatty liver  Alcohol induced chronic pancreatitis  Alcoholic hepatitis  Korsakoff’s psychosis  Wernicke’s encephalopathy | Date, code term, associated text |
| Alcohol referral | e.g. community alcohol team | Date, code term, associated text |
| Audit C score (alcohol) |  | Date, score |
| Smoking | Tobacco use and exposure – finding  Nicotine dependence  Tobacco | Date, code term, associated text, how much |
| Suicide or self-harm attempts | Suicide attempt  H/O: suicide attempt  Suicide  Attempts suicide  Suicide and self-inflicted injury  First known suicide attempt  Injury due to suicide attempt  Self-injurious behaviour  Intentionally harming self  H/O: deliberate self harm | Date, code term, associated text |
| Physical health conditions | Coronary artery disease  Stroke  Hypertension  Atrial fibrillation  Viral hepatitis  HIV  Sexually transmitted disease  COPD  Asthma  Epilepsy  Migraine  Diabetes Mellitus  Cancer  Developmental mental disorder  Rheumatoid arthritis  Ulcers of lower extremity  Peptic ulcer  GI haemorrhage | Date, code term, associated text, problem status |
| PEFR/ FEV1/FVC | Peak expiratory flow rate  Forced expired volume in 1 second  FEV1/FVC  FEV1/FVC ratio | Code term, value, unit of measure |
| Mental health | Mental disorder | Date, code term, associated text |
| Medication | All | Name, dose, quantity, first issue date, number of issues in course, most recent issue date in course, course status, linked problem |
| Criminal justice record | All | Date, code term, associated text |

**Electronic Supplementary Material 3:** Search criteria for substance misuse (excluding alcohol) on EMIS Web

| **Criteria** | **Main branch** | **Children** |  |
| --- | --- | --- | --- |
| Substance misuse | Psychostimulant dependence | Amfetamine and/or amfetamine derivative drug dependence |  |
|  |  | Cocaine dependence |  |
|  |  | Psychostimulant dependence continuous |  |
|  |  | Psychostimulant dependence episodic |  |
|  | Sedative dependence | Benzodiazepine dependence |  |
|  | Methamphetamine dependence |  |  |
|  | Psychoactive substance dependence | Anxiolytic dependence |  |
|  |  | Buprenorphine dependence |  |
|  |  | Fentanyl dependence |  |
|  |  | Hallucinogen dependence |  |
|  |  | Heroin dependence |  |
|  |  | Hypnotic dependence |  |
|  |  | Methadone dependence |  |
|  |  | Morphine dependence |  |
|  |  | Opium dependence |  |
|  |  | Psychostimulant dependence |  |
|  |  | Sedative dependence |  |
|  |  | Synthetic cannabinoid dependence |  |
|  | Opioid dependence | Buprenorphine dependence |  |
|  |  | Combined opioid with other drug dependence |  |
|  |  | Continuous opioid dependence |  |
|  |  | Episodic opioid dependence |  |
|  |  | Fentanyl dependence |  |
|  |  | Heroin dependence |  |
|  |  | Methadone dependence |  |
|  |  | Morphine dependence |  |
|  |  | Opioid analgesic dependence |  |
|  |  | Opioid dependence, on agonist therapy |  |
|  |  | Opium dependence |  |
|  | Stimulant abuse | Abuse of synthetic cathinone |  |
|  |  | Cocaine abuse |  |
|  |  | Harmful pattern of use of amfetamine and/or amfetamine derivative |  |
|  |  | Harmful pattern of caffeine |  |
|  |  | Nondependent psychostimulant abuse |  |
|  | Psychoactive substance abuse | Abuse of antidepressant drug |  |
|  |  | Abuse of synthetic cathinone |  |
|  |  | Cocaine abuse |  |
|  |  | Hallucinogen abuse |  |
|  |  | Harmful pattern of use of amfetamine and/ or amfetamine derivative |  |
|  |  | Harmful use of amphetamine |  |
|  |  | Harmful use of anxiolytic |  |
|  |  | Harmful use of barbiturate |  |
|  |  | Harmful use of cannabis |  |
|  |  | Harmful use of cocaine |  |
|  |  | Harmful use of hallucinogen |  |
|  |  | Harmful use of hypnotic |  |
|  |  | Harmful use of inhalant |  |
|  | Opioid abuse |  |  |
|  | Drug abuse, continuous |  |  |
|  | Abuse of non-psychotropic analgesic drugs |  |  |
|  | Dependent drug abuse |  |  |
|  | Barbiturate abuse |  |  |
|  | Finding related to drug misuse behaviour | Concerned about own drug use |  |
|  |  | Craves for drugs |  |
|  |  | Current drug user |  |
|  |  | Drug injecting behaviour |  |
|  |  | Drug misuse reported by other |  |
|  |  | Drug seeking behaviour |  |
|  |  | Drug-related offending behaviour |  |
|  |  | Misuse of medication |  |
|  |  | Misuse of prescription drugs |  |
|  |  | Misuses drugs |  |
|  |  | Patient admits to drug use |  |
|  |  | Substance misuse of amphetamines |  |
|  |  | Substance misuse of anti-depressants |  |
|  |  | Substance misuse of barbiturates |  |
|  |  | Substance misuse of benzodiazepines |  |
|  |  | Substance misuse of cannabis |  |
|  |  | Substance misuse of cocaine |  |
|  |  | Substance misuse of crack |  |
|  |  | Substance misuse of ecstasy |  |
|  |  | Substance misuse of hallucinogens |  |
|  |  | Substance misuse of heroin |  |
|  |  | Substance misuse of major tranquilisers |  |
|  |  | Substance misuse of methadone |  |
|  |  | Substance misuse of other drugs |  |
|  |  | Substance misuse of other opiates |  |
|  |  | Substance misuse of poly drug |  |
|  |  | Substance misuse of solvents |  |
|  | Misuses drugs | Amphetamine misuse |  |
|  |  | Antidepressant misuse |  |
|  |  | Barbiturate misuse |  |
|  |  | Benzodiazepine misuse |  |
|  |  | Cannabis misuse |  |
|  |  | Cocaine misuse |  |
|  |  | Currently injecting drugs |  |
|  |  | Drug addict |  |
|  |  | Habitual drug user |  |
|  |  | Illicit drug use |  |
|  |  | Inhales drugs |  |
|  |  | Injecting drug user |  |
|  |  | Long term drug misuser |  |
|  |  | Methadone misuse |  |
|  |  | Misuse of analgesic |  |
|  |  | Misuses anabolic steroids |  |
|  |  | Misuses drugs orally |  |
|  |  | Narcotic drug user |  |
|  |  | Nitrous oxide misuse |  |
|  |  | Poly-drug misuser |  |
|  |  | Sniffs drugs |  |

**Electronic Supplementary Material 4:** Search criteria for alcohol misuse on EMIS Web

| **Criteria** | **Main branch** | **Children** |  |
| --- | --- | --- | --- |
| Alcohol misuse | Alcohol abuse | Nondependent alcohol abuse |  |
|  |  | Persistent alcohol abuse |  |
|  | Alcohol dependence syndrome | Alcoholism |  |
|  |  | Mental and behave dis due to use alcohol (all branches) |  |
|  |  | Mild alcohol dependence |  |
|  |  | Moderate alcohol dependence |  |
|  |  | Severe alcohol dependence |  |
|  | Non-dependent alcohol abuse | Non-dependent alcohol abuse in remission |  |
|  |  | Non-dependent alcohol abuse, continuous |  |
|  |  | Non-dependent alcohol abuse, episodic |  |
|  | Alcohol user | Fairly heavy drinker |  |
|  |  | Heavy drinker |  |
|  |  | Moderate drinker |  |
|  |  | Unhealthy alcohol drinking behaviour |  |
|  |  | Very heavy drinker |  |
|  | Alcohol problem drinking | Chronic alcoholism in remission |  |
|  |  | Continuous chronic alcoholism |  |
|  |  | Episodic chronic alcoholism |  |
|  | Alcoholic intake above recommended sensible limits |  |  |
